# The first synapse in vision in the aging mouse retina

**DOI:** 10.3389/fncel.2023.1291054

**Published:** 2023-11-03

**Authors:** Kaspar Gierke, Uwe Thorsten Lux, Hanna Regus-Leidig, Johann Helmut Brandstätter

**Affiliations:** Animal Physiology/Neurobiology, Department of Biology, Friedrich-Alexander-Universität Erlangen-Nürnberg, Erlangen, Germany

**Keywords:** aging, retina remodeling, photoreceptor degeneration, ribbon synapse, mitochondrial alterations, ectopic synapses

## Abstract

Vision is our primary sense, and maintaining it throughout our lifespan is crucial for our well-being. However, the retina, which initiates vision, suffers from an age-related, irreversible functional decline. What causes this functional decline, and how it might be treated, is still unclear. Synapses are the functional hub for signal transmission between neurons, and studies have shown that aging is widely associated with synaptic dysfunction. In this study, we examined the first synapse of the visual system – the rod and cone photoreceptor ribbon synapse – in the mouse retina using light and electron microscopy at 2–3 months, ~1 year, and >2 years of age. We asked, whether age-related changes in key synaptic components might be a driver of synaptic dysfunction and ultimately age-related functional decline during normal aging. We found sprouting of horizontal and bipolar cells, formation of ectopic photoreceptor ribbon synapses, and a decrease in the number of rod photoreceptors and photoreceptor ribbon synapses in the aged retina. However, the majority of the photoreceptors did not show obvious changes in the structural components and protein composition of their ribbon synapses. Noteworthy is the increase in mitochondrial size in rod photoreceptor terminals in the aged retina.

## Introduction

Aging tissue is often faced with a decline in functionality on virtually every aspect of a cell. This includes DNA damage, changes to the metabolism and deterioration of protein homeostasis. Neurons and in particular their synapses, are especially vulnerable to age-related functional decline, leading to irreversible impairment of sensory and cognitive function (reviewed by Mattson and Magnus, 2006). Vision is our primary sense, and its decline affects a significant percentage of individuals during their lifetime. The retina initiates vision and has thus been the subject of studies regarding normal aging in humans and mice (Gao and Hollyfield, 1992; Curcio et al., 1993; Liets et al., 2006; Eliasieh et al., 2007; Kolesnikov et al., 2010; Samuel et al., 2011; Cunea et al., 2014; Samuel et al., 2014).

In healthy humans and mice, the cell classes of the retina are located in distinct layers. With progressing age, the retina starts to lose this precise layering and undergoes structural remodeling. For example, bipolar cells and horizontal cells undergo neurite reorganization, and photoreceptor synapses, normally confined to the outer plexiform layer (OPL), are ectopically located in the outer nuclear layer (ONL) (Liets et al., 2006; Eliasieh et al., 2007; Terzibasi et al., 2009; Samuel et al., 2011). Structural remodeling is accompanied by the dysfunction and/or death of certain types of retinal cells, such as photoreceptors, bipolar cells, or the retinal pigment epithelium, resulting in the functional decline of the retina and impaired visual performance (for reviews see Owsley, 2016; Campello et al., 2021; Zhu et al., 2023). *In vivo* electroretinographic (ERG) recordings suggest that photoreceptor to bipolar signaling is increasingly impaired in the aging retina (Jackson et al., 2002; Specht et al., 2007; Kolesnikov et al., 2010; Freund et al., 2011; Sugita et al., 2020). Similar observations to those in the aging retina were made in retinae lacking key synaptic proteins, e.g., Bassoon (Bsn) or the Ca_v_1.4 L-type Ca^2+^ channel. Removal of these synaptic proteins alone is sufficient to trigger retinal remodeling, ectopic synapse formation, photoreceptor degeneration and the concomitant decline in retinal function, closely resembling the phenotypes observed during normal aging (Mansergh et al., 2005; Specht et al., 2007; Kolesnikov et al., 2010; Kerov et al., 2018; Burger et al., 2021; Ryl et al., 2021). Given this similarity, we asked whether changes during normal aging and synaptopathies share common features that originate from dysfunctional synaptic transmission. For this, we studied specifically the first synapse of the retina – the rod and cone photoreceptor ribbon synapse – in young (2–3 months), adult (~1 year) and aged (>2 years) mice with a focus on the presynaptic compartment, using immunocytochemistry and electron microscopy. We found that key presynaptic components of individual photoreceptor ribbon synapses remained remarkably stable throughout life, but that there was a significant loss of synapses in the OPL in the aged mouse retina. Notably, the size and structure of synaptic mitochondria was compromised in the aged photoreceptors, suggesting their involvement in driving the functional decline of the retina during normal aging.

## Materials and methods

### Animals

Mice were group housed at the animal care facility of the Friedrich-Alexander-Universität Erlangen-Nürnberg (Biologisch-technisches Entwicklungslabor) under a 12-h light/dark cycle with *ad libitum* access to food and water. In this study, wildtype C57BL/6j animals (JAX) were used for analysis. 3–4 male or female mice were analyzed for each age group: 8–12 weeks of age (2–3 months), 60 weeks of age (~1 year) and 117–130 weeks of age (>2 years). Killing of the mice to obtain the retinal tissue was approved by the local authorities (Sachgebiet Tierschutzangelegenheiten der Friedrich-Alexander-Universität Erlangen-Nürnberg, AZ TS 7/2023 Tierphysiologie).

### Antibodies

The following antibodies (primary and secondary) and dyes were used for immunocytochemistry: *Active Zone/ Synaptic Ribbon*: rabbit anti-α1f (Ca_v_1.4) (1:3,000, Synaptic Systems, Göttingen, Germany, cat.no. 365003); mouse anti-Bassoon (1:2,500, Synaptic Systems, cat.no. 141111); rabbit anti-ubMunc13-2 (1:6,000, Cooper et al., 2012); mouse anti-CtBP2 (1:20,000, BD Transduction, Franklin Lakes, NY, USA, cat.no. 612044); *SNARE Complex*: rabbit anti-Complexin4 (1:40,000, Synaptic Systems, cat.no. 122402); mouse anti-Synaptotagmin1 (1:1,000, Synaptic Systems, cat.no. 105011); rabbit anti-Syntaxin3 (1:2,500, Synaptic Systems, cat.no. 110003); *Synaptic Vesicles*: rabbit anti-Rab3A (1:2,000, Synaptic Systems, cat.no. 124003); mouse anti-Synaptophysin (1:10,000, Synaptic Systems, cat.no. 101011); guinea pig anti-vGluT1 (1:50,000, Chemicon, cat.no. AB 5905); *Endocytosis*: mouse anti-Clathrin light chain (1:1,000, Synaptic Systems, cat.no. 113011); rabbit anti-Dynamin3 (1:10,000, Synaptic Systems, cat.no. 115302); rabbit anti-Endophilin (1:10,000, Synaptic Systems, cat.no. 159002); *Calcium Homeostasis*: rabbit anti-MPP4 (1:5,000, Synaptic Systems, cat.no. 220103); mouse anti-PSD95 (1:20,000, abcam, Cambridge, UK, cat.no. ab 18258); rabbit anti-Velis3 (1:5,000, Thermo Fisher Scientific, cat.no. 51–5600); *Retinal Cell Types*: rabbit anti-guinea pig anti-Calbindin D-28 k (1:2,000, Synaptic Systems, cat.no. 214005); Cone Arrestin (1:3,000, Merck Millipore, Burlington, MA, USA, cat.no. AB15282); mouse anti-PKCα (1:20,000, BD Transduction, cat.no. AB397514); DAPI (0.1 μg/mL, Thermo Fisher Scientific, Waltham, MA, USA, cat.no. D1306). Primary antibodies were visualized by suitable Alexa or Cy Dye-coupled secondary antibodies according to the manufacturer’s instruction.

### Light microscopy and immunocytochemistry

Preparation of retinal tissue was performed according to established protocols (Dick et al., 2003). Briefly, mice were anesthetized by inhalation of isofluorane and killed by cervical dislocation. Next, the cornea was removed along the *ora serrata* before removing lens and vitreous body. Retinae were fixed for 15 min in 4% paraformaldehyde in 0.01 M PBS and incubated in rising sucrose series (10 – 30%) before mounting in Tissue TEK O.C.T. for cryosectioning. Retinae were cut vertically into 14 μm thick sections with a cryostat (CM3050 S, Leica Microsystems, Wetzlar, Germany). Primary antibody incubation was performed overnight at 4°C, secondary antibody incubation for 2 h at room temperature. Specimens were mounted in AquaPoly/Mount (PolySciences, Warrington, PA, USA). For analysis, labeled sections were imaged using an Axio Imager M2 equipped with an ApoTome.2 (both Carl Zeiss Microscopy GmbH, Oberkochen, Germany). Images were taken with a 20 × objective (0.8 NA, Apochromat) or a 63 × objective (1.4 NA oil immersion, Plan Apochromat) as z-stacks of multiple optical sections. The *quantification of fluorescence intensities and measurements of AZ and SR areas* were performed with a semi-automatic approach as follows: images were taken with identical exposure times and Z-stacks of five optical sections were projected onto a single plane by maximum image projection (ImageJ, NIH). Images were transformed into binary images and analyzed via the “analyze particle” function in ImageJ, yielding the AZ and SR areas as a proxy for their length. Binary images further served as a mask, in which fluorescence intensities in the OPL were measured in the non-binary images. Measured gray values were normalized to the mean fluorescence intensity of all samples per microscope slide. Normalized mean fluorescence intensities were averaged across three animals per age group (2–3 months and >2 years). *Cone photoreceptor terminal numbers* were manually counted along the OPL and converted to the unit “cone photoreceptors per mm OPL.” *Cone photoreceptor outer segment length* was quantified manually by tracing intact outer segments in vertical cryostat sections labeled with Cone Arrestin. Outer segments (OS) were quantified only if they were clearly discernable from weaker labeled inner segments and if they were intact from the preparation procedure. *Outer nuclear layer thickness measurements* were performed by manual analysis of vertical cryostat sections labeled with the nuclear marker DAPI. Because cone photoreceptor density and ONL thickness varies across the retina, all quantifications were performed in sections from within 100 μm of the optic nerve to ensure comparability. Images were arranged using CorelDRAW 2021 (Corel Corporation, Ottawa, ON, Canada).

### Electron microscopy

Good tissue preservation (“best fix”) of retinal tissue was performed as described previously (Müller et al., 2019). Briefly, retinae were fixed in 4% paraformaldehyde and 2.5% glutaraldehyde in 0.1 mol L^−1^ PB buffer for 2 h at room temperature, followed by postfixation in 2% osmium tetraoxide for 1.5 h at room temperature. After dehydration in rising ethanol concentrations (30 – 100%) and a 5 min incubation step in propylene oxide, retinae were embedded in Epoxy resin (Fluka, Buchs, Switzerland). For analysis, ultrathin sections were cut with an Ultracut E microtome (Reichert-Jung, Nussloch, Germany). Finally, samples were contrasted with lead citrate and uranyl acetate in an automated contrasting system (EM AC20, Leica Microsystems, Wetzlar, Germany). Sixty nm thick ultrathin sections were examined with an EM10 transmission electron microscope (Carl Zeiss AG) equipped with a SC1000 OriusTM CCD camera (Gatan, Pleasanton, CA, USA) in combination with the DigitalMicrograph 3.1 software (Gatan). For the *quantification of the SR dimensions (height, width), SR shapes, SV pools (reserve pool, SR-tethered pool), SV diameter, and mitochondrial area*, images of rod and cone photoreceptor terminals were taken at random locations along the OPL. The dataset used for all analyses contained 150 images from *n* = 3 animals acquired at 25000 × magnification.

### Statistical analysis

All statistical analysis and graph generation was performed using GraphPad Prism 10 (GraphPad Software Inc., San Diego, CA, USA). For statistical analysis, we calculated mean values from individual data points for each animal. The calculated mean values for each animal per age group were compared with each other either using Student’s *t*-test (two groups) or one-way ANOVA (>2 groups). Corrections were applied when data did not follow a normal distribution (indicated in the respective figure legends).

## Results

### Outer retina remodeling and ectopic ribbon synapses in the aged mouse retina

**Figure 1:**
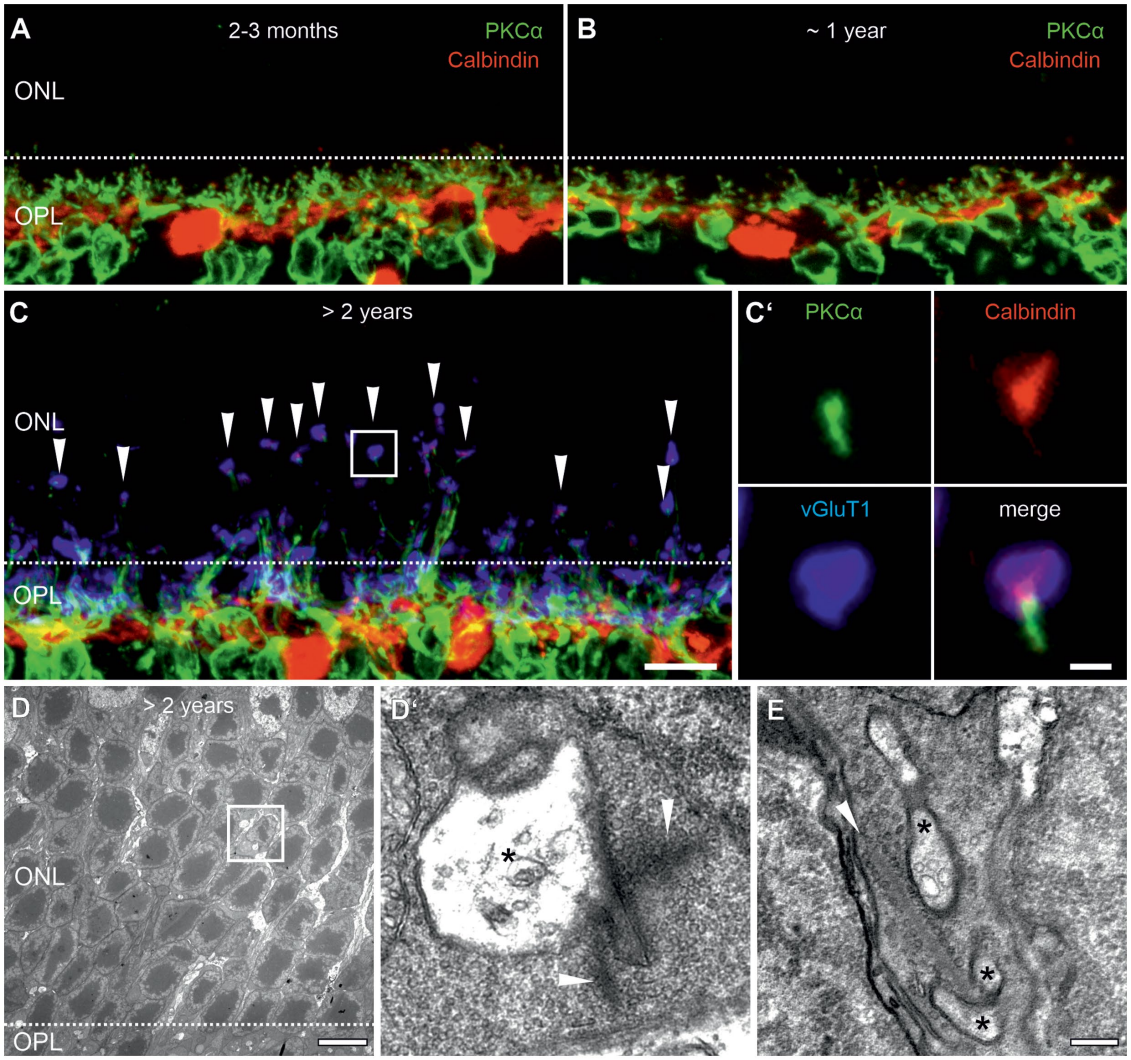
Outer retina remodeling and ectopic ribbon synapses in the aged mouse retina. **(A,B)** Vertical cryosections of a 2–3 months **(A)** and a ~1 year old **(B)** retina labeled for rod bipolar cells (PKCα, green) and horizontal cells (Calbindin, red). **(C)** Vertical cryosection of a >2 years old retina labeled for rod bipolar cells (PKCα, green), horizontal cells (Calbindin, red), and photoreceptor terminals (vGluT1, blue, arrowheads). Boxed area marks an ectopic ribbon synapse in the outer nuclear layer (ONL) shown in higher magnification in **(C’)**. **(D)** Low magnification electron micrograph of the ONL of a >2 years old mouse retina with an ectopic ribbon synapse (boxed area). Dashed line marks the border between the outer plexiform layer (OPL) and ONL. **(D’)** Higher magnification of the boxed area shows a presynaptic ribbon (arrowheads) contacting electron-lucent postsynaptic elements (asterisks). **(E)** Another example of an ectopic ribbon synapse with a presynaptic ribbon contacting postsynaptic elements (asterisks). Scale bar in **(C)** for **(A–C)** = 10 μm; in **(C’)** = 1 μm; in **(D)** = 0.5 μm; in **(E)** for **(D,E)** = 0.2 μm.

We studied aging processes in the outer retina and photoreceptor ribbon synapses of mice starting at 2 months of age, when ribbon synapses have matured (Blanks et al., 1974; Regus-Leidig et al., 2009; Davison et al., 2022) until more than 2 years of age, when functional decline has been reported (Kolesnikov et al., 2010; Samuel et al., 2011). We first investigated the sprouting of neurites of rod bipolar cells (RBCs) and horizontal cells (HCs), the postsynaptic neurons of photoreceptors, into the outer nuclear layer (ONL) and the formation of ectopic ribbon synapses as a sign of aging, degeneration, and outer retinal remodeling. Retinae of 2–3 months, ~1 year and >2 years old mice were labeled with markers for RBCs (PKCα, green) and HCs (Calbindin, red) (Figures 1A–C′). Consistent with previous studies in mouse (Liets et al., 2006; Terzibasi et al., 2009; Samuel et al., 2011) and human retina (Eliasieh et al., 2007), we found that in young (2–3 months) and adult (~1 year) retinae, processes of HCs and dendrites of RBCs were mostly confined to the outer plexiform layer (OPL), the region where photoreceptors usually form ribbon synapses with their postsynaptic partners (Figures 1A,B). This changed in aged retinae (>2 years), where labeling was no longer restricted to the OPL, but RBCs and HCs extended neurites into the ONL (Figure 1C). Additional labeling of presynaptic rod and cone photoreceptor terminals for vGluT1 (blue), demonstrated that these neurites contact photoreceptor terminals at ectopic ribbon synapses in the ONL (Figure 1C′). The presence of ectopic ribbon synapses was further confirmed by electron microscopy. Examples are shown in Figures 1D,E, where presynaptic ribbons contact postsynaptic processes, most likely HC processes, in ectopic locations in the ONL.

### Loss of rod photoreceptors in the aged mouse retina

**Figure 2:**
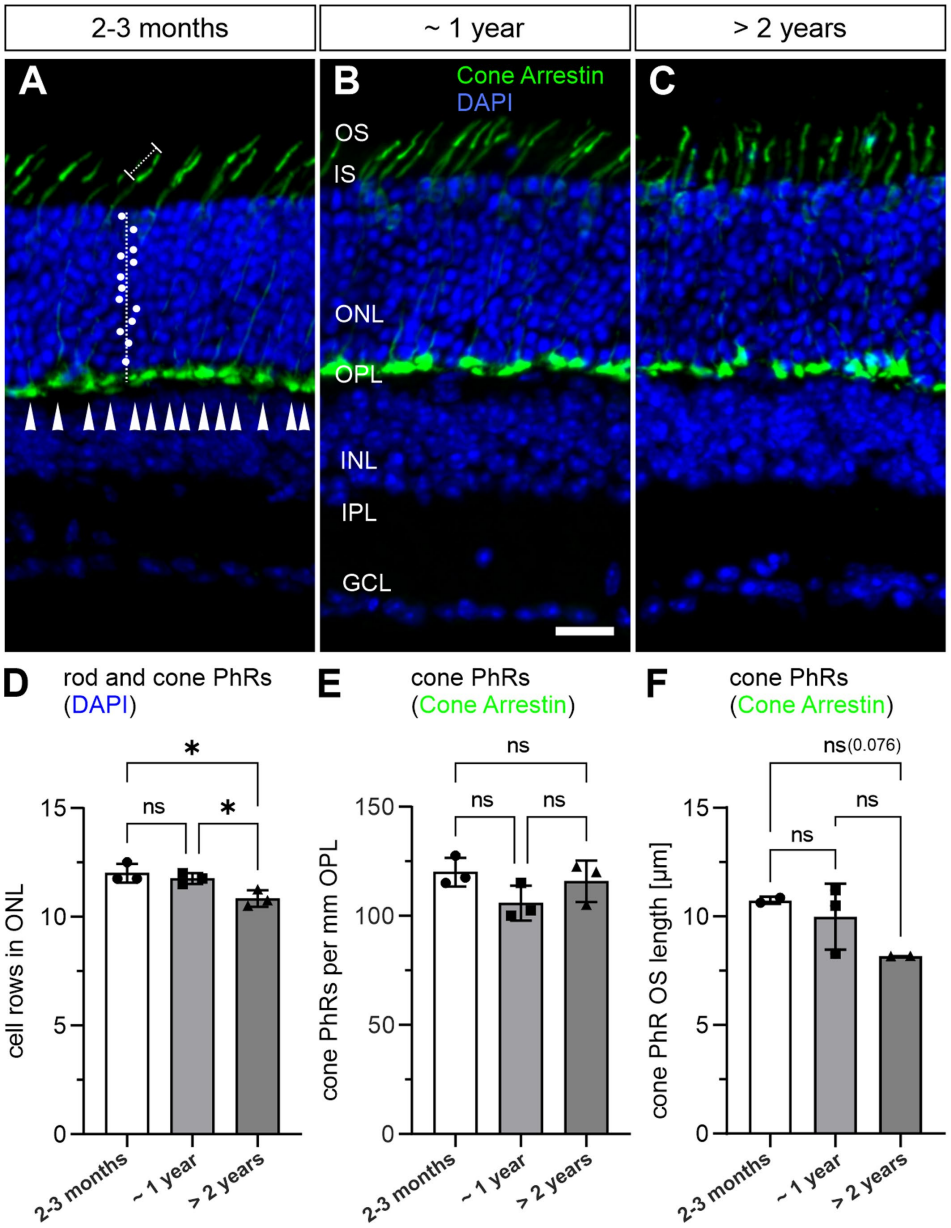
Loss of rod photoreceptors in the aged mouse retina. **(A–C)** Vertical cryosections of a 2–3 months old **(A)**, a ~1 year old **(B)** and a >2 years old mouse retina **(C)** labeled with the nuclear marker DAPI (blue) and the cone photoreceptor marker PNA (green). White dots mark rod and cone photoreceptor nuclei counted along a vertical line through the outer nuclear layer (ONL). Arrowheads mark cone photoreceptor terminals along the outer plexiform layer (OPL). Dotted line marks individual cone photoreceptor outer segments (OS). **(D)** Quantification of rod and cone photoreceptors by DAPI labeling in 2–3 months, ~1 year and > 2 years old retinae. **(E)** Quantification of cone photoreceptors by PNA labeling in 2–3 months, ~1 year and >2 years old retinae. **(F)** Quantification of cone photoreceptor OS. Data collected from *n* = 6 sections from *n* = 3 animals (except cone photoreceptor OS quantification: *n* = 2 for 2–3 months and >2 years old retinae) and compared by one-way ANOVA. n.s., not significant; *p < 0.05. IS, inner segment; INL, inner nuclear layer; IPL, inner plexiform layer; GCL, ganglion cell layer. Scale bar in **(B)** for **(A–C)** = 20 μm.

We next investigated whether photoreceptor degeneration occurs during normal aging. As a readout for the total rod and cone photoreceptor population, we quantified the number of cell rows in the ONL by visualizing photoreceptor nuclei with DAPI staining in 2–3 months, ~1 year and >2 years old retinae (Figures 2A–C). We observed no significant change in cell row numbers between 2–3 months and ~1 year of age (12.0 vs. 11.8 cell rows; *p* = 0.69). However, we found a slight, albeit significant decrease in cell row number in >2 years old retinae (10.8 cell rows; *p* = 0.018) (Figure 2D). As cone photoreceptors represent only about 3% of the total photoreceptor population in the mouse retina (Jeon et al., 1998), we quantified them separately by labeling with the well-established marker Cone Arrestin (Zhang et al., 2003; Figures 2A–C). Cone photoreceptor density was similar between the investigated age groups and showed no obvious trend towards lower numbers when comparing 2–3 months, ~1 year and >2 years old retinae (2–3 months: ~120 cone photoreceptors/mm OPL, ~1 year: ~105 cone photoreceptors/mm OPL, >2 years: ~116 cone photoreceptors/mm OPL; *p* = 0.81) (Figures 2A–C,E; Table 1). Because OS degeneration has been shown to precede cell death (Fisher et al., 2005; Meschede et al., 2020), we additionally measured OS length of cone photoreceptors in 2–3 months, ~1 year and >2 years old retinae. OS length was similar between 2–3 months and ~1 year of age (10.74 μm vs. 9.99 μm; *p* = 0.47). A decrease in OS length was observed in >2 years old retinae (8.17 μm) but did not reach statistical significance (*p* = 0.076) (Figures 2A–C,F). Taken together, these findings suggest that while rod photoreceptors become more vulnerable with age, cone photoreceptors stay resilient, a finding previously reported in aged human retina (Nag, 2021).

**Table 1:** Quantification of cellular and synaptic parameters of aging rod and cone photoreceptors.

**Rod and cone photoreceptor cell numbers**					
**Parameter**		**Unit**	**2–3 months old**	**>2 years old**	***p*** **value**
Rod PhRs (DAPI)		Cell rows	12.0	10.8	0.018
Cone PhRs (Cone Arrestin)		nr. per mm	120	116	0.81
Cone PhR OS length (Cone Arrestin)		μm	10.74	8.17	0.076
**Rod and cone photoreceptor AZ and SR dimensions**					
AZ area		μm^2^	1.37	1.50	0.52
AZ number		nr. per 100 μm	173	149	0.039
SR area		μm^2^	1.43	1.14	0.40
SR number		nr. per 100 μm	159	110	< 0.001
SR height (rod PhRs)		nm	253	259	0.54
SR height (cone PhRs)		nm	230	232	0.86
SR width (rod PhRs)		nm	41.2	41.1	0.92
SR width (cone PhRs)		nm	43.3	43.6	0.83
Rod PhR terminals + SR		%	44	46	0.89
Cone PhR terminals + SR		%	75	75	0.94
**Rod and cone photoreceptor SV diameter and SV pool size**					
SV reserve pool (rod PhRs)		nr. per μm^2^	277	255	0.59
SV reserve pool (cone PhRs)		nr. per μm^2^	216	204	0.75
SV tethered pool (rod PhRs)		nr. per μm^2^	18.9	19.7	0.75
SV tethered pool (cone PhRs)		nr. per μm^2^	19.3	20.7	0.56
SV diameter (rod PhRs)		nm	40.6	39.3	0.16
SV diameter (cone PhRs)		nm	44.3	43.7	0.75
**Rod and cone photoreceptor synaptic mitochondria**					
Total mito. Area (rod PhRs)		μm^2^	0.34	0.51	0.047
Single mito. Area (cone PhRs)		μm^2^	0.23	0.27	0.18
Total mito. Area (cone PhRs)		μm^2^	1.21	1.76	0.017
Single mito. Number (cone PhRs)		nr. per cone	5.29	6.54	0.06
**Rod and cone photoreceptor protein quantification**					
Active Zone	Bassoon	AU	1.00	0.84	0.15
	Ca_v_1.4	AU	1.00	1.06	0.33
	ubMunc13-2	AU	1.00	1.42	0.08
SNARE Complex	Syntaxin3	AU	1.00	2.03	< 0.001
	Synaptotagmin1	AU	1.00	0.96	0.98
	Complexin4	AU	1.00	1.81	0.03
Synaptic Vesicles	vGluT1	AU	1.00	1.21	0.79
	Synaptophysin	AU	1.00	1.45	0.09
	Rab3A	AU	1.00	1.16	0.94
Endocytosis	Clathrin	AU	1.00	0.86	0.78
	Endophilin	AU	1.00	1.10	0.74
	Dynamin3	AU	1.00	0.81	0.43
Ca^2+^ Homeostasis	Velis3	AU	1.00	0.96	0.93
	MPP4	AU	1.00	1.11	0.83
	PSD95	AU	1.00	1.24	0.53

PhR, photoreceptor; OS, outer segment; AZ, active zone; SR, synaptic ribbon; SV, synaptic vesicle; AU, arbitrary units.

### Decrease in the number, but not the size of AZs and SRs in the aged photoreceptors

**Figure 3:**
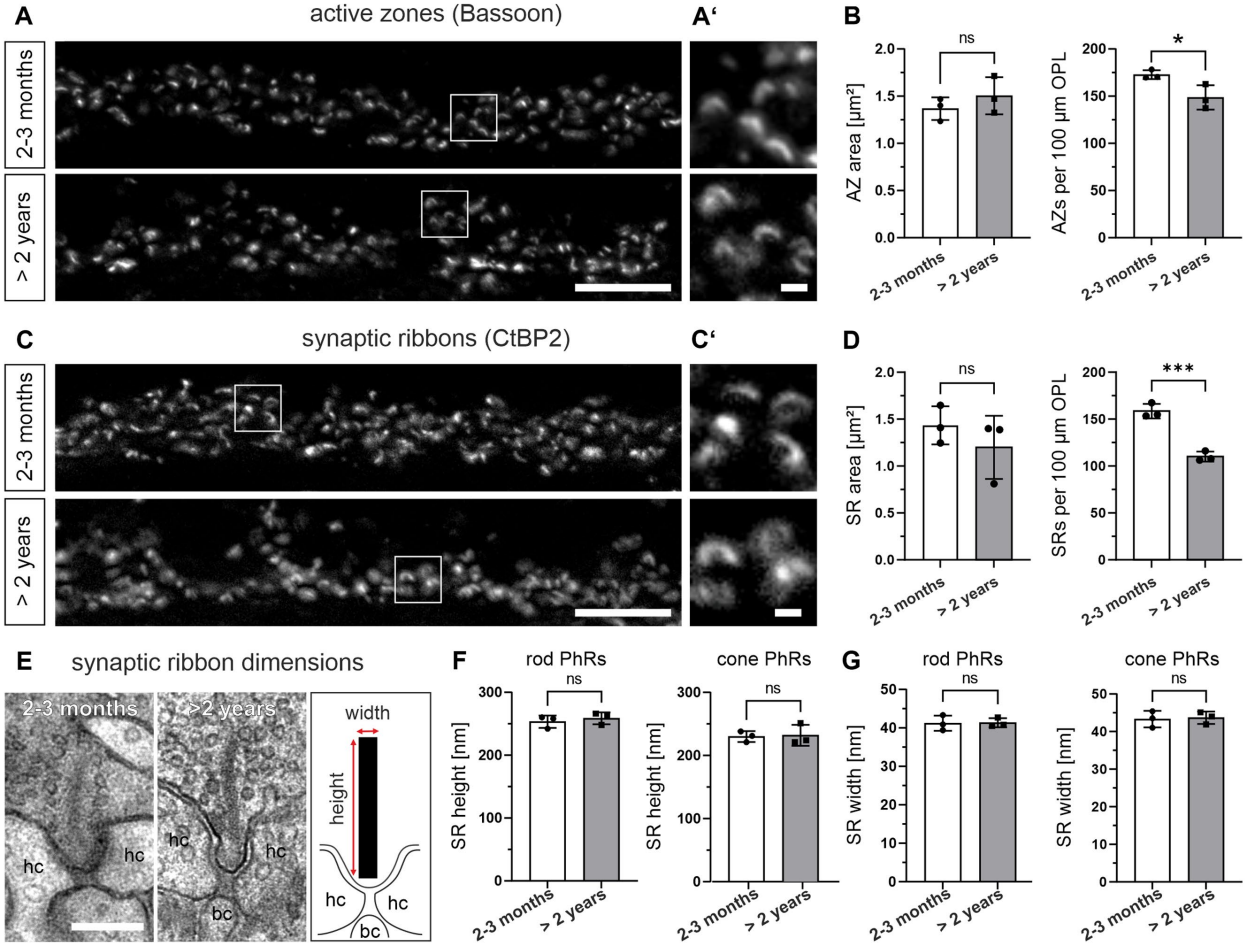
Decrease in the number, but not the size of active zones (AZs) and synaptic ribbons (SRs) in the aged photoreceptors. **(A)** Outer plexiform layer (OPL) of 2–3 months and >2 years old retinae labeled for AZs (Bsn). **(A’)** Higher magnification of the boxed areas in **(A)**. **(B)** Quantification of AZ area and AZ number in 2–3 months and >2 years old retinae. Data collected from *n* = 736 AZs (2–3 months) and *n* = 633 AZs (>2 years) from *n* = 3 animals in each age group. Mean values per animal statistically compared using student’s *t*-test. **(C)** OPL of 2–3 months and >2 years old retinae labeled for SRs (CtBP2). **(C’)** Higher magnification of the boxed areas in **(C)**. **(D)** Quantification of SR area and SR number in 2–3 months and >2 years old retinae. Data collected from *n* = 676 SRs (2–3 months) and *n* = 551 SRs (>2 years) from *n* = 3 animals in each age group. Mean values per animal statistically compared using student’s *t*-test (normal distribution) or Mann–Whitney *U* test (non-normal distribution). **(E)** Representative electron micrographs of SRs in 2–3 months and >2 years old retinae. Ribbon scheme depicts how SR dimensions were measured. **(F,G)** Quantification of SR height **(F)** and SR width **(G)** in 2–3 months and >2 years old retinae. Data collected from *n* = 107 rod SRs and *n* = 28 cone SRs (2–3 months) and *n* = 86 rod SRs and *n* = 20 cone SRs (>2 years) from *n* = 3 animals in each age group. Mean values per animal statistically compared using student’s *t*-test. n.s., not significant; *p < 0.05, ***p < 0.001. bc, bipolar cell; hc, horizontal cell. Scale bar in **(A,C)** = 10 μm; in **(A’,C’)** = 1 μm; in **(E)** = 0.2 μm.

ERG recordings on the aging mouse retina show a decrease in b-wave amplitude at old age (Specht et al., 2007; Kolesnikov et al., 2010), which corresponds to altered photoreceptor to bipolar cell signaling. Since size and number of presynaptic AZs and SRs are crucial determinants of photoreceptor to bipolar cell synaptic signaling (tom Dieck et al., 2012), we next analyzed both parameters during normal aging. We used Bassoon as a marker for AZs (tom Dieck et al., 1998; Brandstätter et al., 1999) and CtBP2 as a marker for SRs (Schmitz et al., 2000) and compared the labeling between 2–3 months and >2 years old retinae (Figures 3A–D; Table 1). The area of AZs did not change significantly during normal aging (2–3 months: 1.37 μm^2^, >2 years: 1.50 μm^2^; *p* = 0.52) (Figures 3A,B). However, the number of AZs counted in the OPL decreased significantly in the aged retina (2–3 months: 173 AZs/100 μm OPL, >2 years: 149 AZs/100 μm OPL; *p* = 0.039) (Figure 3B). Similarly, the area of SRs showed no significant change during normal aging (2–3 months: 1.43 μm^2^, >2 years: 1.14 μm^2^; *p* = 0.40) (Figures 3C,D), while SR number counted in the OPL decreased significantly in the aged retina (2–3 months: 159 SRs/100 μm OPL, >2 years: 110 SRs/100 μm OPL; *p* < 0.001) (Figure 3D). To also account for synaptic terminals without SRs, we performed a quantitative electron microscopic analysis and screened synaptic terminals for the presence or absence of SRs in single ultrathin sections. Our analysis revealed no significant differences in the number of SRs in the OPL for either rod photoreceptors (2–3 months: SRs in 44% of all rod photoreceptor terminals, >2 years: SRs in 46% of all rod photoreceptor terminals; *p* = 0.89) or cone photoreceptors (2–3 months: SRs in 75% of all cone photoreceptor terminals, >2 years: SRs in 74% of all cone photoreceptor terminals; *p* = 0.94) (Table 1).

We next analyzed the height and the width of SRs during normal aging by electron microscopy (Figures 3E–G). Measurements of SR height in single ultrathin sections showed no age-related differences for either rod photoreceptor SRs (2–3 months: ~253 nm, >2 years: ~259 nm; *p* = 0.54) or cone photoreceptor SRs (2–3 months: ~230 nm, >2 years: ~232 nm; *p* = 0.86) (Figure 3F). Similarly, measurements of SR width in single ultrathin sections did not reveal significant differences for either rod photoreceptor SRs (2–3 months: 41.2 nm, >2 years: 41.4 nm; *p* = 0.92) or cone photoreceptor SRs (2–3 months: 43.3 nm, >2 years: 43.6 nm; *p* = 0.83) (Figure 3G) in normal aging. In addition to the dimensions of the SRs, we also examined their shape. In photoreceptors (2–3 months and > 2 years old), 100% of the SRs in cone photoreceptors and >90% of the SRs in rod photoreceptors were plate-shaped (data not shown). Taken together, no significant age-related changes in the dimensions and shape of rod and cone photoreceptor SRs were observed.

### Synaptic vesicle pools are not altered in the aged photoreceptors

**Figure 4:**
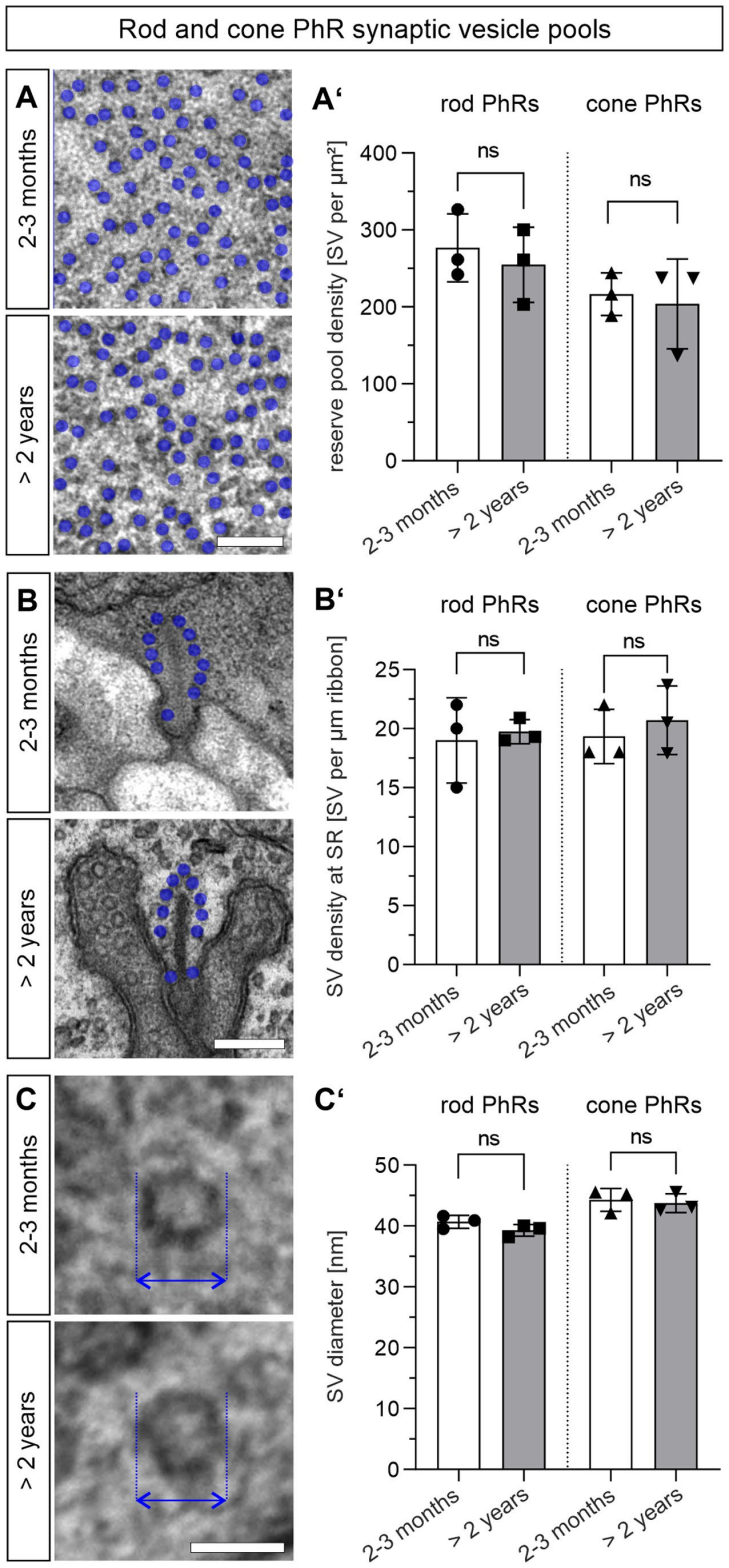
Synaptic vesicle pools are not altered in the aged photoreceptors. **(A,A’)** Quantification of the SV reserve pool density in 2–3 months and >2 years old rod and cone photoreceptor terminals. SVs (blue dots) counted in a randomly placed 0.25 μm × 0.25 μm square in randomly selected rod and cone photoreceptor terminals. Data collected from *n* = 30 synaptic terminals from *n* = 3 animals in each age group. Mean values per animal statistically compared using student’s *t*-test. **(B,B’)** Quantification of SR-tethered SVs in 2–3 months and >2 years old mouse rod and cone photoreceptor terminals. SVs (blue dots) counted along individual SRs. Data collected from *n* = 30 SRs from *n* = 3 animals in each age group. Mean values per animal statistically compared using student’s *t*-test. **(C,C’)** Quantification of SV diameter (arrows) in 2–3 months and >2 years old rod and cone photoreceptor terminals. Data collected from *n* = 30 synaptic terminals from *n* = 3 animals in each age group. Mean values per animal statistically compared using student’s *t*-test. n.s., not significant. Scale bar in **(A,B)** = 0.2 μm; in **(C)** = 0.05 μm.

Besides the number, size, and shape of presynaptic AZs and SRs, changes in SVs may contribute to the decline in synaptic transmission in normal aging. We divided the SVs according to their location in the presynaptic terminals into two pools: (i) the readily releasable pool = tethered SVs that are located within one SV diameter along the SR and (ii) the reserve pool = SVs in the cytosol of the photoreceptor terminal that are more than one SV diameter away from the SR. We counted the number of SR-tethered SVs and reserve pool SVs in single ultrathin sections, and compared them between 2–3 months and >2 years old photoreceptors (Figure 4; Table 1). There was no significant difference in the number of SVs in the reserve pool between the two age groups for either rod photoreceptors (2–3 months: ~277 SVs/μm^2^, >2 years: ~255 SVs/μm^2^; *p* = 0.59) or cone photoreceptors (2–3 months: ~216 SVs/μm^2^, >2 years: ~204 SVs/μm^2^; *p* = 0.75) (Figures 4A,A′). The same applies to the density of SR-tethered SVs (rod photoreceptors: 2–3 months: 18.9 SVs/μm, >2 years: 19.7 SVs/μm; *p* = 0.75; cone photoreceptors: 2–3 months: 19.3 SVs/μm, >2 years: 20.7 SVs/μm; *p* = 0.56) (Figures 4B,B′). Finally, we measured the diameter of SVs in single ultrathin sections. As with the number of SVs, no significant difference was found in the diameter of SVs between 2–3 months and >2 years old rod photoreceptors (2–3 months: 40.6 nm, >2 years: 39.3 nm; *p* = 0.16) or cone photoreceptors (2–3 months, 44.3 nm, >2 years old: 43.7 nm; *p* = 0.72) (Figures 4C,C′). Taken together, neither the size of the SV pools nor the diameter of the SVs was affected during normal aging.

### Photoreceptor presynaptic proteins are preserved in the aged photoreceptors

**Figure 5:**
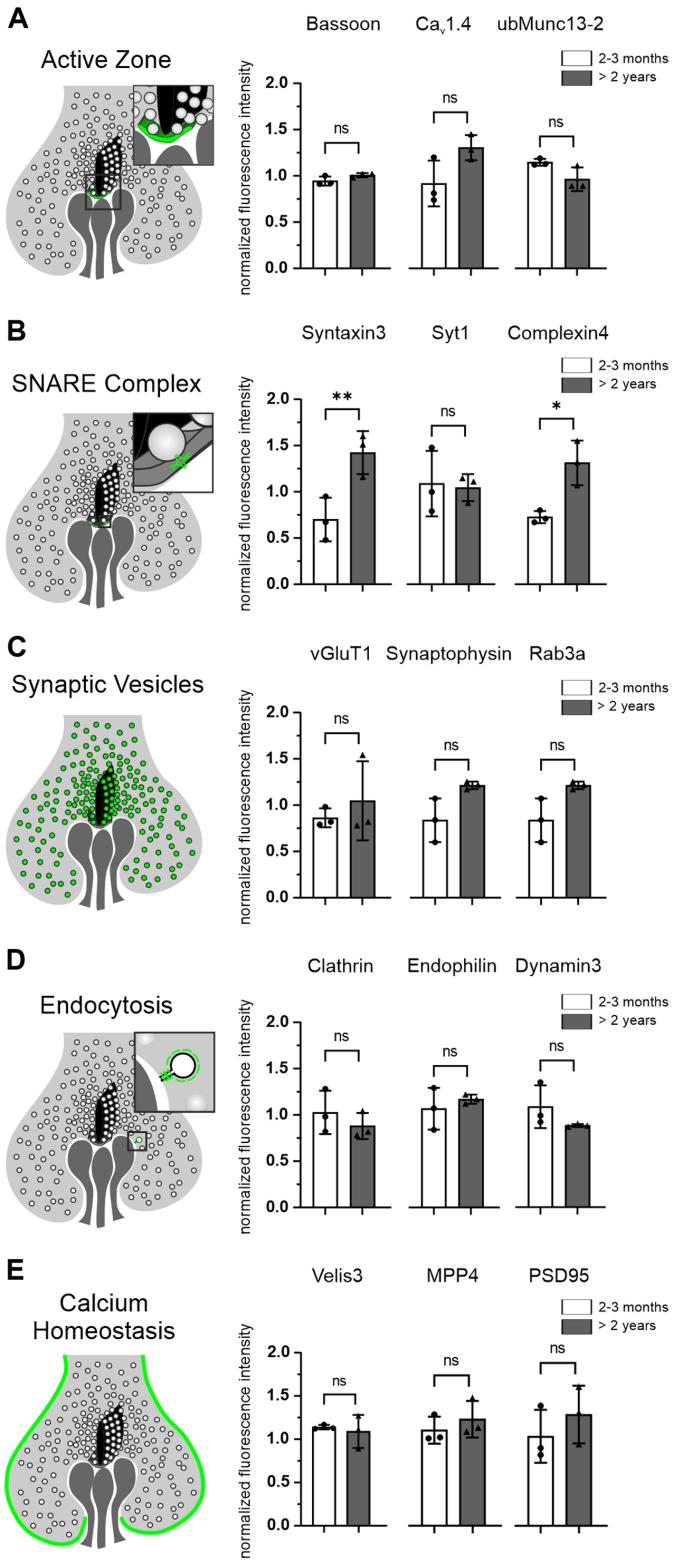
Photoreceptor presynaptic proteins are preserved in the aged photoreceptors. **(A–E)** Schematic of photoreceptor terminals and the localization of the 5 groups of presynaptic proteins analyzed, each combined with the quantification of their fluorescence intensities in 2–3 months and >2 years old photoreceptors. Data collected from *n* = 6 sections from *n* = 3 animals in each age group. Mean values per animal statistically compared using one-way ANOVA. n.s., not significant; *p < 0.05, **p < 0.01.

Aging cells are often faced with a dysregulated proteostasis, leading to either the loss or the aggregation of proteins. To investigate whether key proteins of the presynaptic compartment of photoreceptor ribbon synapses are affected during normal aging, we measured their fluorescence intensity as a semi-quantitative measure of protein levels and compared the fluorescence intensities between 2–3 months and >2 years old photoreceptors. For comparison, all images were taken with the same exposure time. We analyzed 5 groups of proteins, 3 proteins within each group, representing different functional components of the presynapse (Figure 5; Table 1): AZ proteins Bassoon, Ca_v_1.4, ubMunc13-2; SNARE complex and SNARE regulatory proteins Syntaxin3, Synaptotagmin1, Complexin4; SV proteins vGluT1, Synaptophysin, Rab3a; endocytosis proteins Clathrin, Endophilin, Dynamin3; Ca^2+^ homeostasis proteins Velis3, MPP4, PSD95. Of the 5 groups of functionally distinct presynaptic proteins, only the SNARE complex and SNARE regulatory proteins Syntaxin3 (*p* < 0.001) and Complexin4 (*p* = 0.03) showed significant age-related changes, i.e., an increase in fluorescence intensity with age (Figure 5B). The majority of presynaptic proteins showed no age-related changes in fluorescence intensity as a read-out of protein levels during normal aging (Figure 5).

### Rod photoreceptor synaptic mitochondria are enlarged in the aged photoreceptors

**Figure 6:**
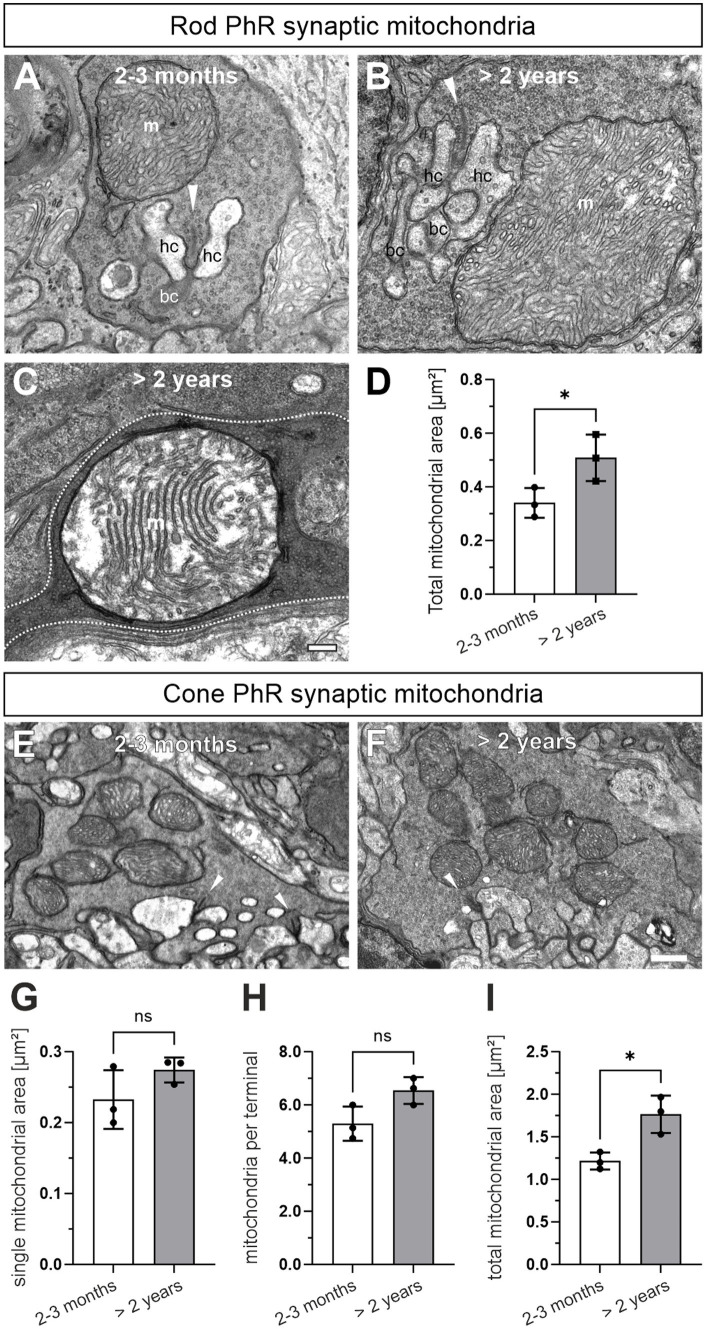
Rod photoreceptor synaptic mitochondria are enlarged in the aged photoreceptors. **(A)** Electron micrograph of a 2–3 months old rod photoreceptor ribbon synapse with adjacent mitochondrion (m). Arrowhead points at synaptic ribbons. **(B,C)** Representative electron micrograph of a >2 years old rod photoreceptor ribbon synapse with enlarged mitochondrion **(B)** and enlarged mitochondrion with disintegrated cristae **(C)**. Dashed line in **(C)** marks synaptic terminal outline. **(D)** Quantification of the total synaptic mitochondrial area in rod photoreceptors. **(E,F)** Representative electron micrographs of a 2–3 months old **(E)** and a >2 year old cone photoreceptor ribbon synapse **(F)** with adjacent mitochondria. **(G–I)** Quantification of single mitochondrial profile area **(G)**, mitochondrial number **(H)** and total synaptic mitochondrial area **(I)** in cone photoreceptor terminals. Arrowhead points at SRs. Data collected from *n* = 321 rod photoreceptors and *n* = 55 cone photoreceptors (2–3 months) and *n* = 357 rod photoreceptors and *n* = 52 cone photoreceptors (>2 years) from *n* = 3 animals in each age group. Mean values per animal statistically compared using student’s *t*-test. n.s., not significant, *p < 0.05. bc, bipolar cell; hc, horizontal cell. Scale bar in **(C)** for **(A–C)** = 0.2 μm and 0.5 μm in **(F)** for **(E,F)**.

During our ultrastructural studies, we frequently observed rod photoreceptor synaptic mitochondria that were enlarged and/or showed signs of degeneration, i.e., disintegrated cristae, in >2 years old photoreceptors (Figures 6A–C). To confirm this quantitatively, we manually traced the outlines of synaptic mitochondrial profiles in single ultrathin sections and compared their area between 2–3 months and >2 years old rod and cone photoreceptors. Rod photoreceptor terminals contain a single mitochondrion, which showed a significant age-related increase in the mitochondrial area (2–3 months: 0.35 μm^2^, >2 years, 0.50 μm^2^; *p* = 0.048) (Figure 6D). Cone photoreceptor terminals contain several smaller synaptic mitochondria. The individual mitochondrial profile also showed a tendency towards increased mitochondrial area, but did not reach statistical significance (2–3 months: 0.23 μm^2^, >2 years: 0.27 μm^2^; *p* = 0.18) (Figures 6E–G). Similarly, the number of mitochondrial profiles increased slightly, but did not reach statistical significance with age (2–3 months: 5.3/cone terminal, >2 years: 6.54/cone terminal; *p* = 0.058) (Figure 6H). A combination of individual area and number of mitochondrial profiles, which yields the total mitochondrial area, showed a significant increase in cone photoreceptor synaptic terminals (Figure 6I). Taken together, enlargement of mitochondria appears to be a hallmark of normal retinal aging especially in rod photoreceptors.

## Discussion

The aging retina undergoes cell-type-specific structural and functional changes that lead to a decline in visual function such as reduced visual acuity, reduced light and contrast sensitivity, or altered motion sensitivity and color perception (for reviews see Owsley, 2016; Campello et al., 2021; Zhu et al., 2023). In this study, we examined the aging mouse retina with a special focus on the first synapse in vision, the photoreceptor ribbon synapse. We studied the retina at 2–3 months of age, when photoreceptor ribbon synapses have matured, and up to more than 2 years of age, when functional retinal decline has manifested. However, as normal aging is a nonlinear process that affects each individual differently, we acknowledge that the relatively small n of three animals per age group might be viewed as a limitation of our study.

### Outer retinal remodeling in the aging mouse retina

We report the sprouting of HC and BC neurites into the ONL and the formation of ectopic photoreceptor ribbon synapses in the ONL in the normal aging mouse retina. Sprouting of HC and BC neurites has been seen as a temporary phenomenon during early postnatal retinal development (Huckfeldt et al., 2009; Regus-Leidig et al., 2014), and it is found in the aged retina. The aging phenotype is a late-onset phenotype, as the changes were only observed in aged mice (Figure 1). Our results are consistent with previous studies in mouse and human retina (Liets et al., 2006; Eliasieh et al., 2007; Terzibasi et al., 2009; Samuel et al., 2011). A key role in the remodeling of the outer retinal network play the rod photoreceptors, which retract their axons from the OPL into the ONL in the normal aging retina. Their postsynaptic partners, HCs and rod BCs, may either adhere to the rod photoreceptor terminals and be pulled into the ONL, or they may grow to the source of glutamate released from the mislocalized rod photoreceptor terminals in the ONL (Samuel et al., 2014; Zhu et al., 2023). To date, the only proteins that have been causally implicated in the remodeling of the rod photoreceptor/rod bipolar cell network in the normal aging mouse retina are the serine/threonine kinase LKB1 and its substrate AMPK. Their absence leads to retinal defects in young mice similar to those described in aged mice (Samuel et al., 2014). Cone photoreceptors in mouse retina appear to be spared from age-related changes in the morphology of their axons, i.e., no retraction from the OPL, and their postsynaptic cone BC partners do not sprout (Samuel et al., 2014). Consistent with this, we found no sprouting of cone BC dendrites in the normal aging mouse retina (data not shown). Interestingly, in the aging human retina changes in cone photoreceptor morphology have been reported (Pow and Sullivan, 2007; Nag et al., 2019; Nag, 2021).

In many pathological conditions, caused by mutations in synaptic proteins, retinal remodeling, particularly sprouting of HC and BC neurites and ectopic ribbon synapse formation, is a common feature (Dick et al., 2003; Haeseleer et al., 2004; Specht et al., 2007; Michalakis et al., 2013; Albert-Fort et al., 2014; Regus-Leidig et al., 2014; Maxeiner et al., 2016; Ryl et al., 2021). A distinguishing factor between the retinal changes due to synaptic protein mutations and aging is time: in mutant mice, the cellular changes occur early in life, whereas in normal aging they occur later in life.

### Loss of photoreceptors and photoreceptor ribbon synapses in the aging mouse retina

Photoreceptors have high metabolic demands and are chronically exposed to light, so it is not surprising that photoreceptor loss is another hallmark of normal retinal aging. In human retina, rod photoreceptors are particularly susceptible to aging, whereas cone photoreceptors appear less vulnerable and show little or no age-related decline in numbers (Gao and Hollyfield, 1992; Curcio et al., 1993). The results from studies in mouse retina are largely in line with those from human retina (Gresh et al., 2003; Williams and Jacobs, 2007; Kolesnikov et al., 2010). However, there is a study reporting the loss of cone photoreceptors in the mouse (Cunea et al., 2014). Our results show the selective loss of rod but not cone photoreceptors in the aging mouse retina (Figure 2). One explanation for the discrepancy between our results and those of Cunea et al. (2014) could be that cone photoreceptor loss was found in M/L expressing cone photoreceptors in the peripheral ventral mouse retina (Cunea et al., 2014), a retinal region not separately examined in our study.

ERG recordings show that photoreceptor-to-BC signaling is increasingly impaired in the aging retina (Jackson et al., 2002; Specht et al., 2007; Kolesnikov et al., 2010; Freund et al., 2011; Sugita et al., 2020). The reduction of the b-wave in the ERG indicates that vision is already affected at the level of the first synapse of the retina, the rod and cone photoreceptor ribbon synapse. To understand the functional decline of aging photoreceptor ribbon synapses, it is essential to understand the changes in synaptic structure. We examined the size, number, and shape of structural components of the photoreceptor presynapse, such as the AZ, the SR, and SVs, and the expression levels of key presynaptic proteins (Figures 3–5; Table 1). To our surprise, we found no significant changes in most of the presynaptic components and proteins examined between the retina of young and aged mice. However, we consider the possibility that a broader approach, e.g., a proteomic analysis, might unveil dysregulated synaptic proteins that we might have missed with our immunostaining approach. Nevertheless, from the findings that the majority of photoreceptor ribbon synapses in the OPL are stable into old age, we conclude that structural changes at individual photoreceptor ribbon synapses do not contribute significantly to the functional decline of mouse retinal vision during normal aging. To ultimately answer this, pre- and postsynaptic recordings from photoreceptors and their postsynaptic partners would be required.

Finally, we found a significant decrease in the number of SRs in the OPL of the aged mouse retina (Figure 3D). Because our quantitative electron microscopic analysis revealed no significant differences in the number of SRs between individual young and aged rod or cone photoreceptor terminals in the OPL (Table 1), we conclude that the loss of SRs from the OPL is caused by rod photoreceptor axon retraction from the OPL into the ONL (Figure 1) and rod photoreceptor degeneration (Figure 2).

### Rod photoreceptor synaptic mitochondria are enlarged in the aging mouse retina

The retina is one of the highest energy consuming tissues of the body (Yu and Cringle, 2001; Wong-Riley, 2010). Mitochondria produce the required ATP via oxidative phosphorylation and are important regulators of Ca^2+^ homeostasis. Both functions are particularly important at retinal photoreceptor ribbon synapses, which require large amounts of energy to maintain sustained neurotransmitter release, and are constantly faced with changes in presynaptic Ca^2+^, and both functions are impaired with age. In their synaptic terminals, rod photoreceptors harbor a single, large mitochondrion and cone photoreceptors multiple, smaller mitochondria close to the site of neurotransmitter release. We found a significant increase in the area of individual mitochondrial profiles and/or signs of mitochondrial disintegration in rod but not cone photoreceptor synaptic terminals in >2 years old retinae (Figure 6). The increase in area most likely represents mitochondrial swelling, which has been observed in several tissues, including the retina, suffering from aging or degeneration (reviewed by Javadov et al., 2018; Zhu et al., 2018).

Recent studies of the aging and degenerating retina show that mitochondrial genes are among the most dysregulated, leading to increased mitochondrial stress and damage (Corso-Diaz et al., 2020; Jiang et al., 2022). Because we did not find major changes in other synaptic components, i.e., AZ, SR, SV pools (Figures 3, 4), one interpretation is that synaptic mitochondrial dysfunction may be a driver of the decline in photoreceptor performance and the loss of rod photoreceptors.

Taking these findings into account raises the question as to why rod photoreceptors, but not cone photoreceptors, degenerate with age (but see Cunea et al., 2014). This is especially puzzling, because cone photoreceptors have been shown to require more energy than rod photoreceptors, and should thus be more sensitive to mitochondrial dysfunction (Okawa et al., 2008; Ingram et al., 2020). We can only speculate that the synaptic mitochondria of cone photoreceptors, because of their smaller size and greater number, are better able to cope with aging than the single large synaptic mitochondrion of rod photoreceptors. In fact, studies have shown that one mechanism that mitigates mitochondrial dysfunctions is an increase in fusion and fission events. These events allow mitochondria to exchange material, such as DNA, and thus reduce the accumulation of damage (Kam and Jeffery, 2015; Hayes et al., 2021). A detailed molecular and functional comparison of aged rod and cone photoreceptor mitochondria might show their selective contribution to photoreceptor survival.

## Data availability statement

The raw data supporting the conclusions of this article will be made available by the authors, without undue reservation.

## Ethics statement

The animal study was approved by Sachgebiet Tierschutzangelegenheiten der Friedrich-Alexander-Universität Erlangen-Nürnberg. The study was conducted in accordance with the local legislation and institutional requirements.

## Author contributions

KG: Data curation, Formal analysis, Investigation, Validation, Visualization, Writing – original draft, Writing – review & editing. UL: Data curation, Formal analysis, Investigation, Validation, Writing – review & editing. HR-L: Conceptualization, Data curation, Formal analysis, Funding acquisition, Investigation, Project administration, Validation, Writing – review & editing, Visualization. JHB: Conceptualization, Project administration, Writing – original draft, Writing – review & editing.

## References

[ref1] Albert-Fort M. Hombrebueno J. R. Pons-Vazquez S. Sanz-Gonzalez S. Diaz-Llopis M. Pinazo-Duran M. D. (2014). Retinal neurodegenerative changes in the adult insulin receptor substrate-2 deficient mouse. Exp. Eye Res. 124, 1–10. doi: 10.1016/j.exer.2014.04.018, PMID: 24792588

[ref2] Blanks J. C. Adinolfi A. M. Lolley R. N. (1974). Synaptogenesis in the photoreceptor terminal of the mouse retina. J. Comp. Neurol. 156, 81–93. doi: 10.1002/cne.901560107 4836656

[ref3] Brandstätter J. H. Fletcher E. L. Garner C. C. Gundelfinger E. D. Wässle H. (1999). Differential expression of the presynaptic cytomatrix protein bassoon among ribbon synapses in the mammalian retina. Eur. J. Neurosci. 11, 3683–3693. doi: 10.1046/j.1460-9568.1999.00793.x, PMID: 10564375

[ref4] Burger C. A. Jiang D. Mackin R. D. Samuel M. A. (2021). Development and maintenance of vision's first synapse. Dev. Biol. 476, 218–239. doi: 10.1016/j.ydbio.2021.04.001 33848537 PMC8559589

[ref5] Campello L. Singh N. Advani J. Mondal A. K. Corso-Diaz X. Swaroop A. (2021). Aging of the retina: molecular and metabolic turbulences and potential interventions. Annu Rev Vis Sci 7, 633–664. doi: 10.1146/annurev-vision-100419-114940, PMID: 34061570 PMC11375453

[ref6] Cooper B. Hemmerlein M. Ammermüller J. Imig C. Reim K. Lipstein N.. (2012). Munc13-independent vesicle priming at mouse photoreceptor ribbon synapses. J. Neurosci. 32, 8040–8052. doi: 10.1523/JNEUROSCI.4240-11.2012, PMID: 22674279 PMC6620942

[ref7] Corso-Diaz X. Gentry J. Rebernick R. Jaeger C. Brooks M. J. Van Asten F.. (2020). Genome-wide profiling identifies DNA methylation signatures of aging in rod photoreceptors associated with alterations in energy metabolism. Cell Rep. 31:107525. doi: 10.1016/j.celrep.2020.107525, PMID: 32320661 PMC7228806

[ref8] Cunea A. Powner M. B. Jeffery G. (2014). Death by color: differential cone loss in the aging mouse retina. Neurobiol. Aging 35, 2584–2591. doi: 10.1016/j.neurobiolaging.2014.05.012, PMID: 24929970

[ref9] Curcio C. A. Millican C. L. Allen K. A. Kalina R. E. (1993). Aging of the human photoreceptor mosaic: evidence for selective vulnerability of rods in central retina. Invest. Ophthalmol. Vis. Sci. 34, 3278–3296. PMID: 8225863

[ref10] Davison A. Gierke K. Brandstätter J. H. Babai N. (2022). Functional and structural development of mouse cone photoreceptor ribbon synapses. Invest. Ophthalmol. Vis. Sci. 63:21. doi: 10.1167/iovs.63.3.21, PMID: 35319739 PMC8963661

[ref11] Dick O. Tom Dieck S. Altrock W. D. Ammermüller J. Weiler R. Garner C. C.. (2003). The presynaptic active zone protein bassoon is essential for photoreceptor ribbon synapse formation in the retina. Neuron 37, 775–786. doi: 10.1016/S0896-6273(03)00086-2, PMID: 12628168

[ref12] Eliasieh K. Liets L. C. Chalupa L. M. (2007). Cellular reorganization in the human retina during normal aging. Invest. Ophthalmol. Vis. Sci. 48, 2824–2830. doi: 10.1167/iovs.06-1228, PMID: 17525218

[ref13] Fisher S. K. Lewis G. P. Linberg K. A. Verardo M. R. (2005). Cellular remodeling in mammalian retina: results from studies of experimental retinal detachment. Prog. Retin. Eye Res. 24, 395–431. doi: 10.1016/j.preteyeres.2004.10.004, PMID: 15708835

[ref14] Freund P. R. Watson J. Gilmour G. S. Gaillard F. Sauve Y. (2011). Differential changes in retina function with normal aging in humans. Doc. Ophthalmol. 122, 177–190. doi: 10.1007/s10633-011-9273-2, PMID: 21562738

[ref15] Gao H. Hollyfield J. G. (1992). Aging of the human retina. Differential loss of neurons and retinal pigment epithelial cells. Invest. Ophthalmol. Vis. Sci. 33, 1–17. PMID: 1730530

[ref16] Gresh J. Goletz P. W. Crouch R. K. Rohrer B. (2003). Structure-function analysis of rods and cones in juvenile, adult, and aged C57bl/6 and Balb/c mice. Vis. Neurosci. 20, 211–220. doi: 10.1017/S0952523803202108, PMID: 12916741

[ref17] Haeseleer F. Imanishi Y. Maeda T. Possin D. E. Maeda A. Lee A.. (2004). Essential role of Ca2+−binding protein 4, a Cav1.4 channel regulator, in photoreceptor synaptic function. Nat. Neurosci. 7, 1079–1087. doi: 10.1038/nn1320, PMID: 15452577 PMC1352161

[ref18] Hayes M. J. Tracey-White D. Kam J. H. Powner M. B. Jeffery G. (2021). The 3D organisation of mitochondria in primate photoreceptors. Sci. Rep. 11:18863. doi: 10.1038/s41598-021-98409-7, PMID: 34552195 PMC8458444

[ref19] Huckfeldt R. M. Schubert T. Morgan J. L. Godinho L. Di Cristo G. Huang Z. J.. (2009). Transient neurites of retinal horizontal cells exhibit columnar tiling via homotypic interactions. Nat. Neurosci. 12, 35–43. doi: 10.1038/nn.2236, PMID: 19060895 PMC2743396

[ref20] Ingram N. T. Fain G. L. Sampath A. P. (2020). Elevated energy requirement of cone photoreceptors. Proc. Natl. Acad. Sci. U. S. A. 117, 19599–19603. doi: 10.1073/pnas.2001776117, PMID: 32719136 PMC7431031

[ref21] Jackson G. R. Ortega J. Girkin C. Rosenstiel C. E. Owsley C. (2002). Aging-related changes in the multifocal electroretinogram. J. Opt. Soc. Am. A Opt. Image Sci. Vis. 19, 185–189. doi: 10.1364/JOSAA.19.000185, PMID: 11778722

[ref22] Javadov S. Chapa-Dubocq X. Makarov V. (2018). Different approaches to modeling analysis of mitochondrial swelling. Mitochondrion 38, 58–70. doi: 10.1016/j.mito.2017.08.004, PMID: 28802667 PMC5752577

[ref23] Jeon C. J. Strettoi E. Masland R. H. (1998). The major cell populations of the mouse retina. J. Neurosci. 18, 8936–8946. doi: 10.1523/JNEUROSCI.18-21-08936.1998, PMID: 9786999 PMC6793518

[ref24] Jiang K. Mondal A. K. Adlakha Y. K. Gumerson J. Aponte A. Gieser L.. (2022). Multiomics analyses reveal early metabolic imbalance and mitochondrial stress in neonatal photoreceptors leading to cell death in Pde6brd1/rd1 mouse model of retinal degeneration. Hum. Mol. Genet. 31, 2137–2154. doi: 10.1093/hmg/ddac013, PMID: 35075486 PMC9618164

[ref25] Kam J. H. Jeffery G. (2015). To unite or divide: mitochondrial dynamics in the murine outer retina that preceded age related photoreceptor loss. Oncotarget 6, 26690–26701. doi: 10.18632/oncotarget.5614, PMID: 26393878 PMC4694945

[ref26] Kerov V. Laird J. G. Joiner M. L. Knecht S. Soh D. Hagen J.. (2018). Alpha(2)delta-4 is required for the molecular and structural Organization of rod and Cone Photoreceptor Synapses. J. Neurosci. 38, 6145–6160. doi: 10.1523/JNEUROSCI.3818-16.2018, PMID: 29875267 PMC6031576

[ref27] Kolesnikov A. V. Fan J. Crouch R. K. Kefalov V. J. (2010). Age-related deterioration of rod vision in mice. J. Neurosci. 30, 11222–11231. doi: 10.1523/JNEUROSCI.4239-09.2010, PMID: 20720130 PMC2928554

[ref28] Liets L. C. Eliasieh K. Van Der List D. A. Chalupa L. M. (2006). Dendrites of rod bipolar cells sprout in normal aging retina. Proc. Natl. Acad. Sci. U. S. A. 103, 12156–12160. doi: 10.1073/pnas.0605211103, PMID: 16880381 PMC1524926

[ref29] Mansergh F. Orton N. C. Vessey J. P. Lalonde M. R. Stell W. K. Tremblay F.. (2005). Mutation of the calcium channel gene Cacna1f disrupts calcium signaling, synaptic transmission and cellular organization in mouse retina. Hum. Mol. Genet. 14, 3035–3046. doi: 10.1093/hmg/ddi336, PMID: 16155113

[ref30] Mattson M. P. Magnus T. (2006). Ageing and neuronal vulnerability. Nat. Rev. Neurosci. 7, 278–294. doi: 10.1038/nrn1886, PMID: 16552414 PMC3710114

[ref31] Maxeiner S. Luo F. Tan A. Schmitz F. Sudhof T. C. (2016). How to make a synaptic ribbon: ribeye deletion abolishes ribbons in retinal synapses and disrupts neurotransmitter release. EMBO J. 35, 1098–1114. doi: 10.15252/embj.201592701, PMID: 26929012 PMC4868958

[ref32] Meschede I. P. Burgoyne T. Tolmachova T. Seabra M. C. Futter C. E. (2020). Chronically shortened rod outer segments accompany photoreceptor cell death in Choroideremia. PLoS One 15:e0242284. doi: 10.1371/journal.pone.0242284, PMID: 33201897 PMC7671558

[ref33] Michalakis S. Schaferhoff K. Spiwoks-Becker I. Zabouri N. Koch S. Koch F.. (2013). Characterization of neurite outgrowth and ectopic synaptogenesis in response to photoreceptor dysfunction. Cell. Mol. Life Sci. 70, 1831–1847. doi: 10.1007/s00018-012-1230-z, PMID: 23269435 PMC11113940

[ref34] Müller T. M. Gierke K. Joachimsthaler A. Sticht H. Izsvak Z. Hamra F. K.. (2019). A multiple Piccolino-ribeye interaction supports plate-shaped synaptic ribbons in retinal neurons. J. Neurosci. 39, 2606–2619. doi: 10.1523/JNEUROSCI.2038-18.2019, PMID: 30696732 PMC6445989

[ref35] Nag T. C. (2021). Pathogenic mechanisms contributing to the vulnerability of aging human photoreceptor cells. Eye (Lond.) 35, 2917–2929. doi: 10.1038/s41433-021-01602-1, PMID: 34079093 PMC8526740

[ref36] Nag T. C. Kathpalia P. Gorla S. Wadhwa S. (2019). Localization of nitro-tyrosine immunoreactivity in human retina. Ann. Anat. 223, 8–18. doi: 10.1016/j.aanat.2019.01.006, PMID: 30716468

[ref37] Okawa H. Sampath A. P. Laughlin S. B. Fain G. L. (2008). Atp consumption by mammalian rod photoreceptors in darkness and in light. Curr. Biol. 18, 1917–1921. doi: 10.1016/j.cub.2008.10.029, PMID: 19084410 PMC2615811

[ref38] Owsley C. (2016). Vision and aging. Annu Rev Vis Sci 2, 255–271. doi: 10.1146/annurev-vision-111815-114550 28532355

[ref39] Pow D. V. Sullivan R. K. (2007). Nuclear kinesis, neurite sprouting and abnormal axonal projections of cone photoreceptors in the aged and Amd-afflicted human retina. Exp. Eye Res. 84, 850–857. doi: 10.1016/j.exer.2007.01.005, PMID: 17343852

[ref40] Regus-Leidig H. Atorf J. Feigenspan A. Kremers J. Maw M. A. Brandstatter J. H. (2014). Photoreceptor degeneration in two mouse models for congenital stationary night blindness type 2. PLoS One 9:e86769. doi: 10.1371/journal.pone.0086769, PMID: 24466230 PMC3897778

[ref41] Regus-Leidig H. Tom Dieck S. Specht D. Meyer L. Brandstatter J. H. (2009). Early steps in the assembly of photoreceptor ribbon synapses in the mouse retina: the involvement of precursor spheres. J. Comp. Neurol. 512, 814–824. doi: 10.1002/cne.21915, PMID: 19067356

[ref42] Ryl M. Urbasik A. Gierke K. Babai N. Joachimsthaler A. Feigenspan A.. (2021). Genetic disruption of bassoon in two mutant mouse lines causes divergent retinal phenotypes. FASEB J. 35:e21520. doi: 10.1096/fj.202001962R 33811381

[ref43] Samuel M. A. Voinescu P. E. Lilley B. N. De Cabo R. Foretz M. Viollet B.. (2014). Lkb1 and Ampk regulate synaptic remodeling in old age. Nat. Neurosci. 17, 1190–1197. doi: 10.1038/nn.3772, PMID: 25086610 PMC5369022

[ref44] Samuel M. A. Zhang Y. Meister M. Sanes J. R. (2011). Age-related alterations in neurons of the mouse retina. J. Neurosci. 31, 16033–16044. doi: 10.1523/JNEUROSCI.3580-11.2011, PMID: 22049445 PMC3238393

[ref45] Schmitz F. Königstorfer A. Südhof T. C. (2000). Ribeye, a component of synaptic ribbons: a protein's journey through evolution provides insight into synaptic ribbon function. Neuron 28, 857–872. doi: 10.1016/S0896-6273(00)00159-8 11163272

[ref46] Specht D. Tom Dieck S. Ammermüller J. Regus-Leidig H. Gundelfinger E. D. Brandstätter J. H. (2007). Structural and functional remodeling in the retina of a mouse with a photoreceptor synaptopathy: plasticity in the rod and degeneration in the cone system. Eur. J. Neurosci. 26, 2506–2515. doi: 10.1111/j.1460-9568.2007.05886.x, PMID: 17970721

[ref47] Sugita Y. Yamamoto H. Maeda Y. Furukawa T. (2020). Influence of aging on the retina and visual motion processing for optokinetic responses in mice. Front. Neurosci. 14:586013. doi: 10.3389/fnins.2020.586013, PMID: 33335469 PMC7736246

[ref48] Terzibasi E. Calamusa M. Novelli E. Domenici L. Strettoi E. Cellerino A. (2009). Age-dependent remodelling of retinal circuitry. Neurobiol. Aging 30, 819–828. doi: 10.1016/j.neurobiolaging.2007.08.017, PMID: 17920161

[ref49] Tom Dieck S. Sanmarti-Vila L. Langnaese K. Richter K. Kindler S. Soyke A.. (1998). Bassoon, a novel zinc-finger cag/glutamine-repeat protein selectively localized at the active zone of presynaptic nerve terminals. J. Cell Biol. 142, 499–509. doi: 10.1083/jcb.142.2.499, PMID: 9679147 PMC2133055

[ref50] Tom Dieck S. Specht D. Strenzke N. Hida Y. Krishnamoorthy V. Schmidt K. F.. (2012). Deletion of the presynaptic scaffold cast reduces active zone size in rod photoreceptors and impairs visual processing. J. Neurosci. 32, 12192–12203. doi: 10.1523/JNEUROSCI.0752-12.2012, PMID: 22933801 PMC6621541

[ref51] Williams G. A. Jacobs G. H. (2007). Cone-based vision in the aging mouse. Vis. Res. 47, 2037–2046. doi: 10.1016/j.visres.2007.03.023, PMID: 17509638 PMC2049007

[ref52] Wong-Riley M. T. (2010). Energy metabolism of the visual system. Eye Brain 2, 99–116. doi: 10.2147/EB.S9078 PMID: 23226947 PMC3515641

[ref53] Yu D. Y. Cringle S. J. (2001). Oxygen distribution and consumption within the retina in vascularised and avascular retinas and in animal models of retinal disease. Prog. Retin. Eye Res. 20, 175–208. doi: 10.1016/S1350-9462(00)00027-6, PMID: 11173251

[ref54] Zhang H. Cuenca N. Ivanova T. Church-Kopish J. Frederick J. M. Macleish P. R.. (2003). Identification and light-dependent translocation of a cone-specific antigen, cone arrestin, recognized by monoclonal antibody 7G6. Invest. Ophthalmol. Vis. Sci. 44, 2858–2867. doi: 10.1167/iovs.03-0072, PMID: 12824223

[ref55] Zhu Y. Pappas A. C. Wang R. Seifert P. Sun D. Jakobs T. C. (2018). Ultrastructural morphology of the optic nerve head in aged and glaucomatous mice. Invest. Ophthalmol. Vis. Sci. 59, 3984–3996. doi: 10.1167/iovs.18-23885, PMID: 30098187 PMC6082327

[ref56] Zhu J. D. Tarachand S. P. Abdulwahab Q. Samuel M. A. (2023). Structure, function, and molecular landscapes of the aging retina. Annu Rev Vis Sci. 9, 177–199. doi: 10.1146/annurev-vision-112122-020950, PMID: 37196423 PMC10524587

